# Relationship between Asymmetry of Gait and Muscle Torque in Patients after Unilateral Transfemoral Amputation

**DOI:** 10.1155/2018/5190816

**Published:** 2018-03-19

**Authors:** Alicja Rutkowska-Kucharska, Mateusz Kowal, Sławomir Winiarski

**Affiliations:** ^1^Department of Biomechanics, University School of Physical Education in Wroclaw, Wroclaw, Poland; ^2^Department of Physiotherapy, Faculty of Health Science, Wroclaw Medical University, Wroclaw, Poland

## Abstract

Many studies have shown that unilateral transfemoral amputation involves asymmetric gait. Transfemoral amputation leads to muscle atrophy in a tight stump resulting in asymmetry in muscle torque between the amputated and intact limb. This research is aimed at verifying if a relationship between torque values of hip joint flexors and extensors and gait asymmetry in patients with TFA exists. Fourteen adult subjects with unilateral TFA took part in the experiment. Gait symmetry was evaluated based on the ground reaction force (GRF). Measurements of muscle torque of hip flexors and extensors were taken with a Biodex System. All measurements were taken under isokinetic (60°/s and 120°/s) and isometric conditions. The symmetry index of vertical GRF components was from 7.5 to 11.5%, and anterio-posterior GRF from 6.2 to 9.3%. The symmetry index for muscle torque was from 24.3 to 44% for flexors, from 39 to 50.5% for extensors, and from 28.6 to 50% in the flexor/extensor ratio. Gait asymmetry correlated with muscle torque in hip joint extensors. Therapy which enhances muscle torque may be an effective form of patient therapy. The patient needs to undergo evaluation of their muscle strength and have the therapy programme adjusted to their level of muscle torque deficit.

## 1. Introduction

People with unilateral transfemoral amputation (TFA) have lost their knee and ankle joint. Frequent complications observed within the amputated limb are muscle atrophy and decrease in muscle contractions in the thigh stump [1]. These lead to a decrease in the muscle torque which stabilizes the hip joint of the amputated limb [2]. Gait asymmetry is reported in range of motion, stride length and width, time variables of the gait cycle, and component values of ground reaction forces (GRF) [3–5].

Many studies have reported that values of the vertical ground reaction force components (vGRF) of an amputated limb in the support phase are lower than for the intact limb [6–8]. Also, the component of the anterior-posterior ground reaction force (a-pGRF) is 50% smaller in the intact limb [9]. Asymmetrical GRF distribution results from the subject's willingness to protect the amputated limb by decreasing the load. Therefore, subjects with TFA shift their centre of mass (CoM) toward the intact limb, which decreases load on the amputated limb [10]. Prolonged asymmetrical loading was found to be a predictor of atrophy in tight stump muscles, overloading, and degenerative changes [11]. Patients with TFA develop compensatory strategies in order to decrease asymmetry, such as the vaulting strategy or hip hiking [12, 13].

Studies on healthy human gait have shown that gait asymmetry may correlate with muscle torque asymmetry [14]. Research evaluating patients with transtibial amputation (TTA) shows statistical significant differences between muscle torque asymmetry of the intact and amputated limbs, 33 ± 20% for extensors and 22 ± 23% for flexors [15]. Moirenfeld et al. [2] pointed to the existence of 49.7 Nm deficit muscle torque in amputed limbs for extensors and 35.1 Nm for flexors of the hip joint in patients with TTA. Hip muscle torque asymmetry between intact and amputed limb can be assumed to point to gait asymmetry. It is important to decrease the asymmetry reported between muscle strength in hip joints because significant differences between the intact and amputated limb may lead to strain and quicken degenerative changes [16]. The ability to decrease muscle torque asymmetry was described in studies comparing symmetry in torque flexors and extensors of subjects with TFA engaged in sports and those physically inactive. The authors concluded that physical activity improves the strength of the muscles that affect the hip joint [17].

This study evaluated gait asymmetry based on the symmetry index of GRF components. Many research papers have examined this problem [18]. The symmetry index can be a criterion differentiating correct and pathological movement patterns, as well as a tool to evaluate the rehabilitation process [14, 19]. It has been hypothesized that an increase in strength ability of hip joint muscles may improve gait symmetry. This can be obtained by introducing resistance exercises to the rehabilitation process of patients with TFA. The correlation between hip joint muscle strength and asymmetry of kinetic gait variables in people with TFA has not been studied. However, there has been some research on subjects with below-knee amputation. Researchers have shown a correlation between gait asymmetry and muscle torque in patients with TTF [10, 20–22]. We expected to observe the same correlation in patients with TFT. That is why this research is aimed at identifying a relationship between torque values, symmetry of muscle torque of hip flexors and extensors of an intact and amputated limb, and a degree of gait asymmetry in patients with TFA.

## 2. Material and Methods

### 2.1. Recruitment and Inclusion Criteria

Fourteen adult subjects with unilateral TFA (mean age: 46 ± 14 years, mean height: 1.76 ± 09 m, mean body mass: 79.6. ± 18.3 kg) took part in the experiment. All participants were subject to gait analysis, but only eight participated in muscle torque evaluation. Six patients did not participate in muscle torque evaluation because their stump was too short (Table 1). Some were physically active and participated in sports like wheelchair tennis, sitting volleyball, swimming, and body building. The patients' body height, mass, age, and amputation characteristics are presented in Table 1. Prior to the research, all participants were informed about its aim and their ability to terminate participation at any stage without providing a reason. All participants provided written, informed consent. Only adults were selected for the research. All subjects used prostheses every day and did not use any other gait aid device. Each participant had been using a prosthetic limb for six months, minimum. The exclusion criteria of the study were stump or lower limb pain and chronic illnesses, which might have influenced motor organ performance.

The research project was approved by the university ethics committee.

### 2.2. Data Processing

#### 2.2.1. Gait

A 6 m walking distance at a self-selected speed enabled the recording of 3 to 4 complete gait cycles. The protocol was run 6 times. Ground reaction force (GRF) data was collected with the use of two Kistler 9286AA-A plates with the frequency of 1 kHz situated at the centre of a pathway [23, 24].

**Figure 1:**
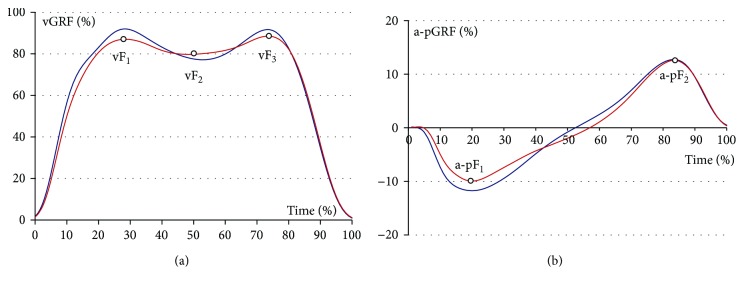
Vertical ground reaction force (vGRF) and anterio-posterior ground reaction force (a-pGRF) components for the amputated (red line) and intact (blue line) limbs with analyzed parameters for the amputated limb.

In addition, mean gait speed was computed for each patient and key moments (heel strike and toe-off) acquired for each measurement trial using SMART-E motion analysis system (BTS Bioengineering, Milan, Italy). Raw GRF measurements were filtered by a 2nd order Butterworth filter with a cut-off frequency of 6 Hz. For the main Cartesian components of GRF vector, such as vertical ground reaction force (vGRF) or horizontal anterior-posterior (a-pGRF) (Figure 1), the researchers conducted a parametrization by computing the following:

- vF_1_: maximal vGRF of overweight at the initial weight acceptance phase
- vF_2_: minimal vGRF of underweight during middle stance
- vF_3_: maximal vGRF of overweight during terminal stance
- a-pF_1_: maximal braking a-pGRF at initial stance
- a-pF_2_: maximal push-off a-pGRF at terminal stance

### 2.3. Torque Measurement

**Figure 2:**
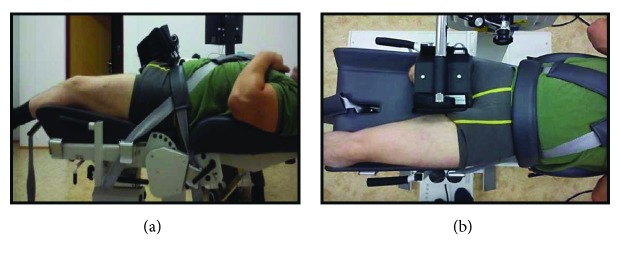
Test position for the hip flexor and extensor muscles. Side and top view.

Measurements of speed-strength abilities in hip joint muscles in flexion (FL) and extension (EXT) were taken with a Biodex System 4 Pro device. The measurement setup was comprised of a chair with an adjustable back angle and seat height, and straps to stabilize the trunk (2 straps) and pelvis (1 strap). An adjustable arm had a strap to stabilize the lower limb or a thigh stump. Prior to measuring, subjects removed their orthopaedic limb and underwent a thigh-skin evaluation of the remaining limb. The subjects' supine position and angular velocities were selected on the basis of the manufacturer's recommendations (Figure 2). All individuals were allowed to familiarize themselves with the type and resistance of movement to be performed. A thigh stump could not be shorter than 22 cm measured from the trochanter. The design of the device enabled the alignment of the dynamometer axis of rotation with the axis of hip-joint movement. All measurements were taken in the sagittal plane under isokinetic (angular velocity 60°/s and 120°/s) and isometric conditions. Flexors (FL) and extensors (EXT) of the hip were studied in sets of 5 repetitions. The time interval between each measurement (rest time) was 1 minute. The following variables were studied: peak torque for FL and EXT under isometric condition, peak torque for FL, and EXT under isokinetic conditions. Ratio of the FL to EXT torques (F/E ratio) for the intact and amputated limbs was calculated. GRF data and muscle torque were normalized to body weight (BW). The following equation was used to compute the symmetry index for gait variables and muscle torque:

(1) $$\begin{array}{c} SI=2\cdot \frac{\left|x_{un}-x_{\text{in}}\right|}{x_{un}+x_{\text{in}}}. \end{array}$$

Symmetry index designates symmetry (low values) or asymmetry (high values) for an *x* variable between uninvolved (un) and involved (amputated) sides and is expressed in percents.

### 2.4. Statistics

Normal distribution of the variables was determined by implementation of the Kolomogorov-Smirnov and Lilliefors tests. Not all values were normally distributed; thus, the Wilcoxon signed-rank test was applied to determine differences between the amputated limb and intact limb. The relationship between muscle torque and gait asymmetry was evaluated with the use of Spearman's rank correlation coefficient. The statistical significance level was set as ɑ = 0.05.

## 3. Results

Gait symmetry was evaluated based on the vertical ground reaction force (vGRF) and the anterior-posterior (a-p GRF) ground reaction force (Table 2). In addition, due to differences observed between patients in regard to their morphological parameters, statistical analysis was carried out for ground reaction forces normalized to body weight (%BW) and normalized muscle torque to body mass (Nm/kg). Variables were statistically smaller for the amputated limb in regard to values of GRF by 7.7%BW (*p* = 0.01) in the support phase (vF_1_), 12.3%BW (*p* < 0.01) in terminal stance (vF_3_), and 12.0%BW (*p* < 0.01) for posterior braking force at initial stance (a-pF_1_). Values of the vertical component of GRF during underweight in middle stance (vF_2_) were on average 5.8%BW higher (*p* < 0.01) for the amputated limb.

Muscle torque of hip joint flexors and extensors in all measurement conditions was statistically significantly lower for the amputated limb (Table 2). Specifically, the differences between the two limbs were 0.28 Nm/kg (*p* = 0.02) for FL and 0.45 Nm/kg (*p* = 0.04) for EXT in isometry, 0.44 Nm/kg (*p* = 0.01) for FL and 0.47 Nm/kg (*p* = 0.01) for EXT in isokinetic 60°/s, and 0.37 Nm/kg (*p* = 0.01) for FL and 0.24 Nm/kg (*p* = 0.05) for EXT in isokinetic 120°/s. There were no statistically significant differences between flexors and extensors (F/E ratio) due to the high variability (high standard deviation).

The next stage of this research was to find correlations between gait symmetry index and muscle torque symmetry index in hip joint flexors and extensors. The analysis showed a positive correlation between the symmetry index of the horizontal GRF in the support phase (vF_1_) and torque symmetry index of hip joint extensors in isometric conditions (Table 3). The remaining gait phases illustrated a correlation of GRF symmetry with the torque symmetry index of hip joint extensors at 120°/s. In this research, no statistically significant differences were found between GRF symmetry and the flexor to extensor ratio (F/E ratio).

Analysis of correlations between gait asymmetry and torque in hip joint flexors (Table 4) showed a statistically significant relationship only in muscles of the amputated limb. The results revealed a positive correlation between the symmetry index in maximal posterior braking a-pGRF at initial stance (a-pF_1_) and muscle torque of hip joint flexors in isometric conditions. A statistically significant correlation between gait asymmetry and muscle torque of the intact and amputated limb extensors was observed for isometric and isokinetic measurements (120°/s). In detail, the muscle torque of extensors obtained in isometric conditions at 120°/s showed a negative correlation with braking force (a-pF_1_) and positive with propulsion force (a-pF_2_) for the amputated limb. There was a negative correlation between muscle torque in isometric conditions and vertical underweight force (vF_2_) observed in the intact limb. Also, a negative correlation was observed between muscle torque in isokinetic conditions at 60°/s and braking force (a-pF_1_) and positive correlation with propulsion force (a-pF_2_). Additional correlations were observed between the F/E ratio. A correlation between the ratio of the amputated limb for maximal muscle isometric force was positive for braking force (a-pF_1_) and negative for propulsion force (a-pF_2_). There was a positive correlation between maximal muscle isometric force and vF_2_ for the intact limb, and a negative correlation between maximal isokinetic force at 60°/s with propulsion force (a-pF_2_).

### 3.1. Individual Results

The subjects differed in terms of age, body mass and height, and level of physical activity. Therefore, there were two statistical analyses conducted by the researchers: one of data collected for the entire group and one of variables related to the individuals. They compared muscle torque in subjects with the results of healthy people at the same age range.

Patient's data was standardized and adjusted individually in respect to age, height, and mass using regression equations provided by Harbo et al. [25].

**Figure 3:**
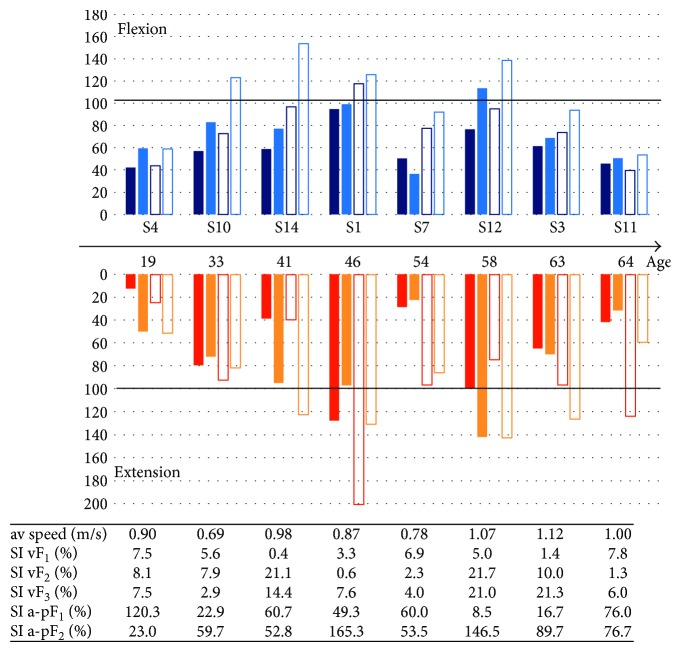
Individual results of the tested subjects: peak torques for flexors and extensors, the average speed (av speed), and symmetry indexes (SI) for GRF variables. Muscle torque values of joint hip flexors and extensors were normalized to values obtained by healthy people in their age category (continuous horizontal line). Dark blue bar denotes the values for isometric conditions and light blue for the isokinetic 60 deg/s conditions for amputated (filled) and intact limb (no fill).

Results obtained by healthy subjects in the selected age groups were normalized to 100% and presented in Figure 3. Patient S1 (46 years old) obtained higher muscle torque values of the hip joint flexor of the intact and amputated limbs than his peers. The same conclusion was drawn for hip joint extensors. Muscle torque values obtained by the intact limb were twice as high, which resulted in great asymmetry between the intact and amputated limb. This patient showed a high level of muscle strength which was related to quite a significant level of gait asymmetry (163.5%) during the anterior push-off a-pGRF at terminal stance. Similar muscle torque was obtained by patient S12 (age 58) who showed greater asymmetry between vertical GRF components.

## 4. Discussion

There are many factors having influence on gait asymmetry in people with unilateral TFA such as age, patient's physical fitness, time post amputation, type of a prosthetic limb, and rehabilitation program. Prolonged time asymmetric loading of the lower limb—intact and amputated—results in atrophy of stump muscles, degenerative changes in the joints of the intact limb, and lower back pain. Consequently, participation in therapy aiming at reducing gait asymmetry seems justifiable. It has been hypothesized that there is a correlation between strength of hip joint muscles and gait asymmetry assessed on the basis of the ground reaction force (GRF) components. Confirmation of this hypothesis was illustrated by a selection of exercises strengthening hip joint muscles in patient therapy [1, 10].

### 4.1. Gait

The typical M-shape observed in healthy people was also characteristic for the vertical (vGRF) component of amputated limbs of the patients [23, 24]. All analyzed GRF variables showed quantitative differences between the amputated and intact limb. They pointed to a significant load on the intact limb, which may in turn cause degenerative changes. This problem has been highlighted by a number of authors [26, 27]. Lower relative GRF values in all gait phases (vF_1_, vF_3_, a-pF_1_, and a-pF_2_) except in the middle stance (vF_2_) were observed for the amputated limb. de Castro et al. [28] observed similar values of a relative vGRF (101.6 for vF_1_ and 97.9 for vF_3_) in a group of patients aged 56.7 ± 11.7 years. However, relative anterior-posterior ground reaction force (a-pGRF) component values assessed by de Castro et al. [28] at initial stance and terminal stance were lower in comparison to our patients (7.12 for a-pF_1_ and 7.4 for a-pF_2_). Schaarschmidt et al. [29] showed similarities to our results of vGRF in the middle stance (in the underweight phase) and terminal stance (in the overweight phase). Furthermore, they concluded that vF_2_ values of an amputated limb decreased along with an increase of gait velocity. Lower vGRF values in the intact limb in the final support phase could occur as an adaptive mechanism to increase the foot clearance of the prosthetic foot, otherwise also known as the vaulting [13]. The results obtained in this research, when compared with those obtained by different authors, showed that vF_1_, vF_3_, and a-pF_1_ GRF of the intact limb were identical for healthy people, while the values for the amputated limbs were much smaller [30, 31].

Research showed that asymmetry in walking over many years with greater loading on the intact limb may be the cause of degenerative changes to weight-bearing joints [32]. Therefore, many authors are interested in gait asymmetry in amputees. This research showed the greatest vGRF asymmetry between the limbs in the terminal stance (vF_3_): 11.5%. Nolan et al. [5] in their research on the relationship between vF_1_ asymmetry and gait velocity (at velocities of 0.5, 0.9, and 01.2 m/s) showed that gait symmetry indices were on average 29.4, 28.9, and 26.0% and were higher than those obtained by our patients (7.5%), presumably, because our patients were physically active. The symmetry index in healthy people depends on gait speed but does not exceed 10% and decreases along with a decrease in speed [5, 33].

### 4.2. Muscle Torque

One of the main causes of gait disturbance in patients after TFA is the imbalance of muscles acting on the hip joint following removal of the femoral ends of major muscles, such as the hamstrings, adductors, rectus femoris, and sartorius muscles. Burger et al. [1] (using electrical stimulation and measurement of muscle belly displacement) illustrated an important function of the gluteus maximus (GM) in improving the quality of gait. They found that atrophy of GM proved by decease of muscle belly displacement of the amputated limb of TFA patients requires programmes of physical therapy directed at strengthening the muscle. These slow down thigh motion at the end of the swing phase and hold the knee joint straight in the support phase [34]. Muscles acting on the hip joint are presumed to have influence on the transfer of weight to the prosthetic limb, similar to people with below-knee amputations [21].

Except for a single case study, we have not found any published research involving torque evaluation of hip joint flexors and extensors in patients with TFA [17]. We had access only to studies regarding patients and these on muscle torque hip abductors [15, 35]. Our research has hypothesized that there is a relationship between gait asymmetry and muscle strength of hip joint flexors and extensors. Evaluation of these muscles was of interest due to the fact that an increased symmetry index of the hip joint flexors and extensors in healthy people causes lower back pain [36]. In this research, relative values of muscle torque of an amputated limb computed for all measurement conditions were significantly lower than those for an intact limb, which resulted in a high value of symmetry index. Relative values of muscle torque of an amputated limb were lower for hip joint flexors than extensors. Also, hip joint extensors were stronger than flexors of an intact limb. An exception was measurements obtained in isokinetic conditions for 120°/s where both groups reached similar relative torque values. There were no statistically significant differences in F/E ratio between intact and amputated limbs. In patients with unilateral TFA, the symmetry index of muscles acting on these joints was quite high. Moreover, the symmetry index computed for flexors increased along with angular velocity (from 24.3% to 43.9%). The opposite behaviour was found for the extensors, and the highest symmetry index was obtained in isometric conditions (50.5 ± 19%). Equally high (50 ± 16%) symmetry indexes between the limbs were observed in the F/E ratio. A similar study was carried out for patients with TTA [15]. The symmetry index between intact and amputated limbs was significantly greater for hip extensors (33% at 30°/s) than flexors (22%). In healthy people, the symmetry index of muscle torque of hip joint flexors and extensors ranges from 1.3 to 5.6% for flexors and 2.3% for extensors [37]. Bae et al. [38], using the electromyography technique (EMG), showed asymmetry in strength of intact and amputated limbs. They concluded that EMG activity in the major muscles for the intact leg was lower than for healthy persons—20.5% for quadriceps and 87.9% for hamstring. But the EMG muscle activities of the tibialis anterior and gastrocnemius were greater than for healthy subjects—14.5% and 15.5%, respectively.

### 4.3. Correlation between Gait Asymmetry and Muscle Torque of Hip Joint Flexors and Extensors

A correlation between gait asymmetry and muscle torque of hip joint muscles in people with TFA was not previously studied. Therefore, it prevents us from comparing our results with those of different authors. Our results showed that a correlation between gait asymmetry and muscle torque exists only for the a-pGRF components. Strong hip flexors and extensors were associated with smaller asymmetry of the anterior-posterior component at the initial stance (a-pF_1_), while weaker flexors and extensors of the amputated limb were associated with smaller asymmetry of the anterior-posterior at the terminal stance (a-pF_2_). This analysis showed a lack of relationship between vertical components of the ground reaction force and muscle torque of hip joint flexors, except in one case. On the other hand, there were many statistically significant correlations between gait asymmetry and muscle torque asymmetry of extensors in regard to an intact and amputated limb.

### 4.4. Case Study Analysis

The group of patients studied was not homogeneous. They differed both in age and physical activity practice. It was observed in terms of muscle torque values of patients with TFA compared to healthy people in the same age. Torque values of the amputated limb obtained in isokinetic conditions (60°/s) for professional athletes (S1, S14, and S12) were similar to or higher than the values for healthy people. Special attention was paid to S1 patient who was a professional athlete in body building. Muscle torque of his hip joint flexors and extensors of the amputated limb was similar to normative values for healthy people, while his intact limb was much stronger, with much higher normative values than those of healthy subjects at the same age (46 years old). Also, the muscle torque value obtained by patient S12 in some conditions was similar or higher than those obtained by healthy people. It can be presumed that therapy through sport activity may improve the mobility of patients with TFA. The study on the positive effect of therapy through sports activity has confirmed greater dynamics in generating maximal muscle torque [17]. Although some tested subjects did not declare sports activity, their muscle torque values were still similar to normative one. A growing body of evidence suggests that muscle torque measurements obtained in isometric conditions should be used in diagnostics of patients with TFA [39].

## 5. Conclusions

This research showed consistent evidence of a significant correlation between hip joint extensors and gait asymmetry. Conclusions drawn on the basis of the study mean group for patients with TFA are limited by errors caused by many factors. We therefore believe that statistical analysis used in such a heterogeneous group may have errors. For this reason, all data for the patients have been included with our analysis. Although statistical analysis showed some correlations, it is the analysis of individual cases that can be useful in improving patients' mobility and quality of life. Sports training which enhances muscle torque may be an effective form of patient therapy. However, prior to commencement, a patient needs to undergo evaluation of their muscle strength and have the therapy programme adjusted to their level of muscle torque deficit.

## Figures and Tables

**Table 1:** Detailed characteristic of patients' body height, mass, age, and type of prosthesis.

Patient	Body height (m)	Body mass (kg)	Age (year)	Side	Cause	Stump length (cm)	Torque	Socket type	Prosthetic knee	Prosthetic foot	Sport activity
P1	1.71	73.2	46	R	Trauma	29.5	+	ICS Anatomica	C-Leg®	1C40 C-Walk®	Body building
P2	2.00	106.0	43	L	Trauma	35.0	+	ICS Anatomica	C-Leg	1C40 C-Walk	_
P3	1.78	96.0	63	L	Trauma	29.0	+	Marlo anatomical socket (MAS®)	3R80®	1C30 Trias®	_
P4	1.83	111.5	19	L	Congenital malformation	34.0	+	ICS Anatomica	3R95®	1C30 Trias	
P5	1.82	93.0	45	R	Trauma	21.0	_	ICS Anatomica	C-Leg	1C40 C-Walk	
P6	1.71	58.3	20	R	Cancer	22.0	_	ICS Anatomica	C-Leg	1C40 C-Walk	Swimming
P7	1.75	81.0	54	L	Trauma	24.0	+	ICS Anatomica	3R80	1C30 Trias	Volleyball
P8	1.68	60.0	36	L	Vascular	27.0	_	ICS Anatomica	3R80	1C30 Trias	_
P9	1.64	46.4	21	L	Cancer	22.5	_	ICS Anatomica	C-Leg	1C60 Triton	Swimming
P10	1.70	80.2	33	L	Congenital malformation	24.0	+	ICS Anatomica	3R95	1C30 Trias	_
P11	1.70	58.1	64	R	Cancer	25.5	_	Marlo anatomical socket (MAS)	3R80	1E56 Axtion®	_
P12	1.83	97.0	58	L	Trauma	32.0	+	ICS Anatomica	C-Leg	1C60 Triton®	Tennis
P13	1.63	59.7	38	R	Trauma	28.5	+	ICS Anatomica	C-Leg	1C60 Triton	_
P14	1.81	93.6	41	R	Cancer	25.0	_	ICS Anatomica	C-Leg	1C40 C-Walk	Volleyball

R: right side; L: left side; +: patients who participated in muscle torque measurement.

**Table 2:** Vertical (v), anterio-posterior (a-p) GRF, and muscle torque of hip flexor and extensor variables and symmetry indexes between sides. Statistically significant differences between the sides are marked with an asterisk.

	Amputated leg	Intact leg	Symmetry index (%)
	Mean ± SD	Mean ± SD	Mean ± SD
GRF (%BW)			
vF_1_	101.9 ± 5.1^∗^	109.6 ± 8.6	7.5 ± 5.4
vF_2_	85.6 ± 5.8^∗^	79.8 ± 8.0	7.8 ± 6.9
vF_3_	95.4 ± 3.1^∗^	107.7 ± 8.8	11.5 ± 10.0
a-pF_1_	8.5 ± 4.7^∗^	20.5 ± 7.5	9.3 ± 4.5
a-pF_2_	10.4 ± 4.1	13.0 ± 6.3	6.2 ± 6.3
Muscle torque (Nm/kg)			
*Flexors*			
Isometric	1.00 ± 0.15^∗^	1.28 ± 0.21	24.3 ± 07.3
Isokinetic 60°/s	0.93 ± 0.15^∗^	1.37 ± 0.21	38.8 ± 15.5
Isokinetic 120°/s	0.77 ± 0.15^∗^	1.14 ± 0.15	43.9 ± 13.9
*Extensors*			
Isometric	1.32 ± 1.44^∗^	1.77 ± 0.65	50.5 ± 19.0
Isokinetic 60°/s	1.11 ± 0.97^∗^	1.58 ± 0.24	39.2 ± 18.4
Isokinetic 120°/s	0.90 ± 0.78^∗^	1.14 ± 0.24	44.8 ± 19.0
F/E ratio			
Isometric	1.05 ± 0.34	0.86 ± 0.26	50.0 ± 16.2
Isokinetic 60°/s	0.93 ± 0.12	0.90 ± 0.11	28.6 ± 08.6
Isokinetic 120°/s	0.98 ± 0.19	1.10 ± 0.19	37.5 ± 11.1

^∗^Significant at *p* < 0.05.

**Table 3:** Correlation coefficients between the symmetry index for ground reaction forces and symmetry index for hip flexor and extensor peak muscle torques for the amputated limb statically and dynamically.

Torque	vF_1_	vF_2_	vF_3_	a-pF_1_	a-pF_2_
*Flexors*					
Isometric	−0.50	0.24	−0.12	−0.29	−0.12
Isokinetic 60°/s	−0.30	−0.07	−0.15	−0.23	−0.05
Isokinetic 120°/s	0.28	−0.10	−0.45	0.40	−0.63^∗^
*Extensors*					
Isometric	0.71^∗^	−0.62	−0.36	0.43	−0.05
Isokinetic 60°/s	0.18	−0.60	−0.22	0.20	0.02
Isokinetic 120°/s	0.68^∗^	−0.73^∗^	−0.67^∗^	0.67^∗^	−0.33
*F/E ratio*					
Isometric	0.67	−0.19	−0.21	0.60	−0.29
Isokinetic 60°/s	0.07	−0.13	−0.15	0.22	−0.20
Isokinetic 120°/s	−0.05	−0.25	−0.62	0.20	−0.33

^∗^Significant at *p* < 0.05.

**Table 4:** Correlation coefficients between the symmetry index for ground reaction forces and hip flexor and extensor muscle peak torques.

Torque	Amputated limb					Intact limb				
	vF_1_	vF_2_	vF_3_	a-pF_1_	a-pF_2_	vF_1_	vF_2_	vF_3_	a-pF_1_	a-pF_2_
						*Flexors*				
Isometric	−0.62	0.07	0.26	−0.71^∗^	0.69	−0.52	0.20	0.32	−0.42	0.40
Isokinetic 60°/s	−0.55	0.10	0.33	−0.60	0.69	−0.60	0.23	0.18	−0.47	0.32
Isokinetic 120°/s	−0.40	0.00	0.21	−0.48	0.67	−0.32	0.07	−0.20	−0.27	0.00
						*Extensors*				
Isometric	−0.38	−0.10	0.26	−0.74^∗^	0.90^∗^	−0.02	−0.73^∗^	−0.43	−0.30	0.53
Isokinetic 60°/s	−0.62	0.33	0.50	−0.60	0.62	−0.65	0.27	0.57	−0.70^∗^	0.77^∗^
Isokinetic 120°/s	−0.55	0.21	0.33	−0.74^∗^	0.71^∗^	−0.38	−0.23	−0.08	−0.47	0.57
						*F/E ratio*				
Isometric	0.31	0.12	−0.02	0.71^∗^	−0.74^∗^	−0.13	0.70^∗^	0.25	0.15	−0.53
Isokinetic 60°/s	0.69	−0.38	−0.67	0.62	−0.67	0.17	0.13	−0.35	0.28	−0.67^∗^
Isokinetic 120°/s	0.79^∗^	−0.60	−0.71^∗^	0.69	−0.52	0.03	0.45	−0.03	0.17	−0.57

^∗^Significant at *p* < 0.05.
